#  Evaluation of anti-urolithiatic effect of aqueous extract of *Bryophyllum pinnatum* (Lam.) leaves using ethylene glycol-induced renal calculi

**Published:** 2014

**Authors:** Apexa Bhanuprasad Shukla, Divyesh Rasikbhai Mandavia, Manish Jasmatbhai Barvaliya, Seema Natvarlal Baxi, Chandrabhanu Rajkishore Tripathi

**Affiliations:** 1 ^*‎*^*Department of Pharmacology, Government Medical College and Sir Takhtsinhji General ‎Hospital, Bhavnagar-364001 (Gujarat), India ‎*; 2 *‎Department of Pathology, Government Medical College and Sir Takhtsinhji General Hospital, ‎Bhavnagar-364001 (Gujarat), India‎*

**Keywords:** *Bryophyllum pinnatum* (*Lam*.) *Calcium oxalate*, *Ethylene glycol*, *Renal calculi*

## Abstract

**Materials and Methods** **:** Thirty-six *Wistar* male rats were randomly divided into six equal groups. Group A animals received distilled water for 28 days. Group B to group F animals received 1% v/v ethylene glycol in distilled water for 28 days and group B served as ethylene glycol control. Groups C and D (preventive groups) received aqueous extract of leaves of *B. pinnatum* 50 and 100 mg/kg intraperitoneally, respectively for 28 days. Groups E and F (treatment groups) received aqueous extract of leaves of *B. pinnatum* 50 and 100 mg/kg intraperitoneally, respectively from 15^th^ to 28^th^ day. On days 0 and 28, 24 hrs urine samples were collected for urinary volume and urinary oxalate measurement. On day 28, blood was collected for serum creatinine and blood urea level monitoring. All animals were sacrificed and kidneys were removed, weighed, and histopathologically evaluated for calcium oxalate crystals deposition.

Results: **Administration of aqueous extract of leaves of B. pinnatum reduced urine oxalate level ‎significantly, as compared with Group B (p<0.001). Serum creatinine and blood urea level were ‎improved significantly in all aqueous extract of leaves of B. pinnatum-treated groups. Relative ‎kidney weight and calcium oxalate depositions were found significantly reduced in animals ‎received ABP as compared with Group B (p<0.001). ‎**

Conclusions: B. pinnatum is effective in prevention and treatment of ethylene glycol-induced urolithiasis.

## Introduction

Renal calculi are common and an extremely painful condition with recurrence rate 70-81% and 47-60% in male and female, respectively (Stoller ML and Bolton DM, 2004 ▶; Manjula K et al., 2012). Renal calculi are the infrequent cause of renal failure even though primary hyperoxaluria, cystinuria, primary struvite stones, and infections with urolithiasis carry a great risk to it (Gambaro G et al., 2001 ▶). 

Recurrent stone formation is also associated with risk of renal damage. Extracorporeal shock wave lithotripsy is widely used for treatment of urolithiasis. Its multiple sessions in recurrent stone formation may cause chronic deterioration of renal function (Kishimoto T et al., 1986 ▶). Currently, there is no established treatment for prevention of urolithiasis. Therefore, there is need to establish a medical treatment for prevention of recurrent stone formation. Indigenous plants have been used as a potential source of medicine since ancient times. Although many plants have been evaluated for anti-urolithiatic effect, search for medical treatment for renal calculi is still going on (Hadjzadeh MA et al., 2007 ▶; Rad AK et al., 2011 ▶). *Bryophyllum pinnatum *(Lam.) commonly known as life plant or air plant is grown in India, China, Australia, New Zealand, and Philippines. 

It is used in traditional medicine as anticancer, antinociceptive, antidiabetic, anti-inflammatory, antifungal, antiulcer, antihypertensive, antimicrobials, hepatoprotective, and antihelminthic (Supratman U et al., 2001 ▶; Ojewole, 2005 ▶; Misra S and Dixit S, 1979 ▶; Pal S et al., 2000 ▶; Ojewole JA, 2002 ▶; Akinpelu DA, 2000 ▶; Yadav NP and Dixit VK, 2003; Majaz QA et al., 2011 ▶). Anti-oxidant and nephroprotective effect of *B. pinnatum *(Lam.) is documented in various studies (Harlalka GV and Patil MR, 2007 ▶; Salah N et al., 1995; Morales AI et al., 2006 ▶). Though it is commonly used as a folk medicine in India to treat renal calculi, scientific evidence to support its effectiveness in renal calculi is lacking. The present study was undertaken to investigate the effect of aqueous extract of *B. pinnatum *(Lam.) leaves on calcium oxalate urolithiasis using ethylene glycol induced renal calculi model of rats and to confirm its medicinal value.

## Material and Methods

The present study was started after ethical approval from Institutional Animal Ethics Committee, Government Medical College, Bhavnagar, Gujarat, India. Male *Wistar* albino rats weighing 250-350 g were obtained from the central animal house of the Institute. Animal care and handling were done according to the Good Laboratory Practice (GLP) guidelines. They were kept in transparent polycarbonate cages at controlled room temperature and humidity (24±2 ^°^C; 65±10%) with maintained 12 hrs dark-light cycle and fed with standard laboratory diet. The animals were allowed to acclimatize for at least five days prior to the experiment. 

Aqueous extract of *B. pinnatum *(ABP) leaves was obtained from Leopard Investments Ltd, Mumbai, India. Doses of ABP were selected as the basis of previous acute toxicity studies. Previous study showed that oral absorption of the active phytoconstituents of this plant is inadequate and erratic (Varma RK et al., 1979 ▶; Varma RK et al., 1986 ▶). Ethylene glycol (Fisher Scientific Co., Mumbai, India), oxalate kit (Trinity Biotech, Ireland), sodium phosphate (Aldrich, India), sodium oxalate, and calcium acetate (Alfa Aesar, Hyderabad, India) were used for the study.

**EG-induced renal calculi**

Thirty-six animals were randomly assigned to six different groups (n=6 each). Total study duration was 28 days. Animals which were given distilled water for 28 days as drinking water *ad libitum* served as normal control (group A) whereas all other groups received 1% v/v ethylene glycol (EG) in distilled water as drinking water for 28 days. Group B served as disease control (sham control). In test groups, ABP was provided in two different doses 50 and 100 mg/kg intraperitoneally for prevention and treatment of renal calculi. In preventive groups (groups C and D), animals received ABP 50 and 100 mg/kg, respectively for 28 days whereas, in treatment groups (groups E and F) animals received 50 and 100 mg/kg, respectively from 15^th^ to 28^th^ day. Administered volume of ABP in each injection was 0.1 to 0.35 ml according to its dose.

On days 0 and 28, animals were kept in separate metabolic cages (B.I.K Industries, Mumbai, India) and 24 hrs urine samples were collected and their volumes were measured. Urine oxalate level was measured using spectrophotometer (Sherwood Model 340 Spectra UV) (Li MG and Madappally MM, 1989 ▶). On 28^th^ day, animals were anesthetized with ketamine (50 mg/kg intraperitoneally) and xylazine (10 mg/kg intra peritoneally) and blood sample was collected from retro-orbital plexus to measure serum creatinine and blood urea level. After blood collection, animals were sacrificed and the abdominal cavity was opened and kidneys were quickly removed, washed with cold saline solution, and weighted. Relative kidney weight (RKW) of each animal was then calculated using the formula: (absolute kidney weight (g)/body weight (g) on day 28) × 100. 

Both kidneys were kept in formaldehyde (10% v/v) for at least 24 hrs. Five-mm sections were processed through a series of graded acetone and xylene, and embedded in paraffin wax. Five-µm thin sections were taken and stained with haematoxylin-eosin (H&E). Calcium oxalate (CaOx) crystal depositions were calculated in consecutive 10 microscopic fields (159×10^-9^ m^2^ each) having homogenous distribution of crystals. Body weight of each animal was measured before and at the end of experiment. Relative body weight (RBW) was calculated using the formula: (absolute body weight (g) on day 28/ body weight (g) on day 0) × 100.

All values were expressed as mean ± standard error of mean (S.E.M.). Urine volume, urine oxalate, serum creatinine, blood urea, RBW, RKW, and CaOx crystal depositions were compared between the groups using one way ANOVA test followed by Tukey-Kramer multiple comparison test as data were normally distributed. The statistical calculations were done using GraphPad InStat (Demo version 3.06). p value<0.05 was considered as significant difference.

## Results

Experimental animals in all of the groups were comparable at the baseline. There was no significant difference for urine volume and oxalate level among the groups on day 0. There was no significant difference found in RBW at the end of the study period between all of the groups. 

**Figure 1:**
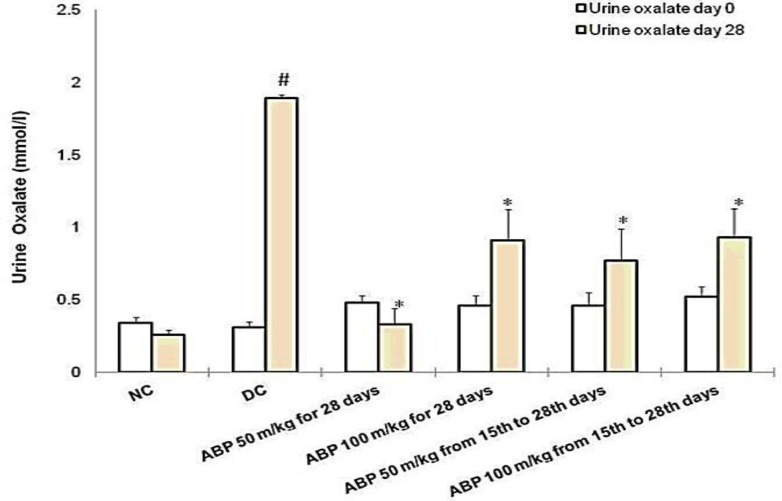
Urine oxalate level at day 0 and day 28. #p<0.001 as compared with NC and *p<0.001 as compared with DC. NC: normal control, DC: disease control, ABP: aqueous extract of *Bryophyllum pinnatum*

**Figure 2:**
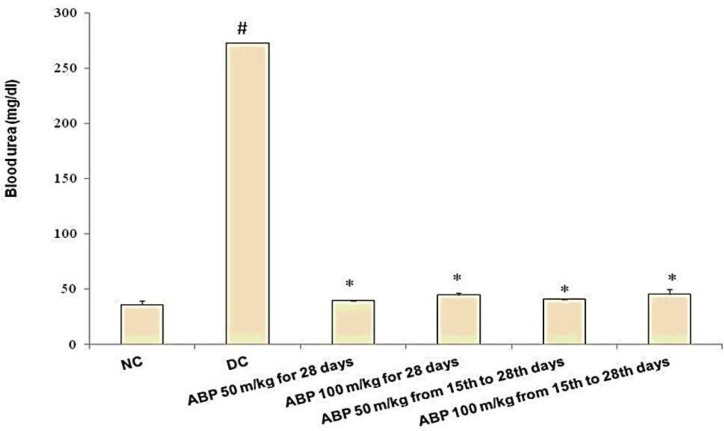
Blood urea level at study end points. # p<0.001 as compared with NC and * p<0.001 as compared with DC. NC: normal control, DC: disease control, ABP: aqueous extract of *B. pinnatum*

**Figure 3:**
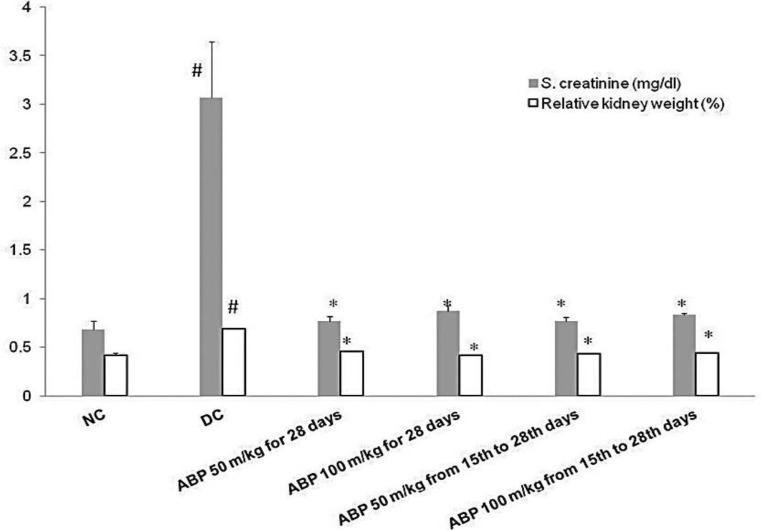
S. creatinine and relative kidney weight at study end points. # p<0.001 as compared with NC and *p<0.001 as compared with DC. NC: normal control, DC: disease control, ABP: aqueous extract of *B. pinnatum*.

**Figure 4:**
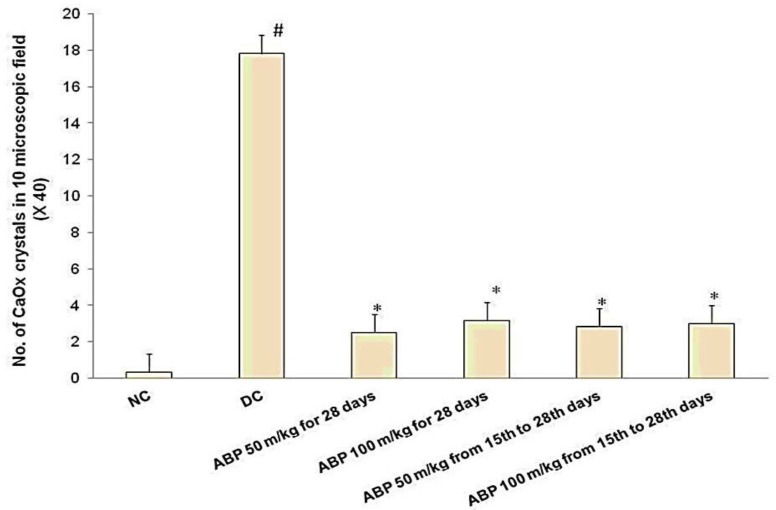
No. of calcium oxalate (CaOx) crystal deposition in 10 microscopic fields (×40) at study end points. #p<0.001 as compared with NC and *p<0.001 as compared with DC. NC: normal control, DC: disease control, ABP: aqueous extract of *Bryophyllum pinnatum*

**Figure 5:**
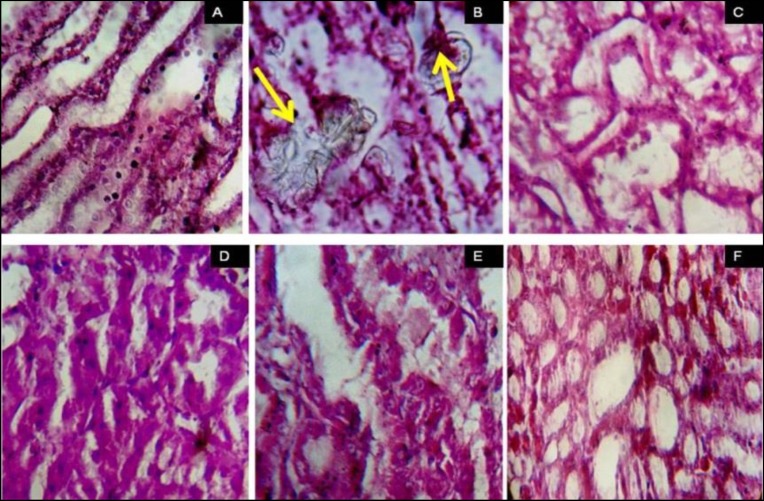
Histopathological images of kidney sections after haematoxylin & eosin staining under light microscope (×40) (a) normal control group, (b) Disease control group showing calcium oxalate crystals (arrows in figure), (C to F) shows aqueous extract of *Bryophyllum pinnatum *(Lam.)-treated groups C to F.

EG 1% v/v-induced renal calculi: In male albino Wistar rats, administration of EG 1% v/v for 28 days successfully induced renal calculi formation. As shown in Figure 1, urine oxalate level was significantly increased compared with normal control group (1.89±0.02 vs. 0.26±0.03, p<0.001) however, urine volume remained unaffected on day 28. Renal function was compromised as evident by increased serum creatinine (3.07±0.57 vs. 0.68±0.09 mg/dl) and blood urea level (272.67±81.33 vs. 36.17±3.44 mg/dl) compared with a normal control group. (p*<*0.001, Figures 2 and 3). As shown in Figure 2, RKW was significantly elevated in the EG-treated group compared with normal control (0.69±0.03 vs. 0.42±0.02 %; p<0.001). EG-treated rats had a significant large number of CaOx crystal deposition in 10 microscopic fields (17.83±1.88 vs. 0.33±0.21) as compared with normal control (p<0.001, Figure 4). CaOx crystal depositions were observed in all parts of kidney with preponderance to renal parenchyma and papillary tips and were associated with degeneration of epithelial lining, tubular dilatation, and necrosis (Figure 5). 

Effect of ABP on EG-induced renal abnormalities: *B. pinnatum *in prophylactic groups (50 and 100 mg/kg) markedly reduced the elevated urinary oxalate concentration due to simultaneous EG administration (0.33±0.11, 0.91±0.21 vs. 1.89±0.02). Similarly in treatment groups, both doses of *B. pinnatum *(0.77±0.22, 0.93±0.2 vs. 1.89±0.02) showed significant reduction in the urinary oxalate concentration on day 28 (p<0.01,Figure 1).

There was no significant effect of *B. pinnatum *on the urinary volume. *B. pinnatum *in both treatment and prophylactic groups showed improvement in renal function tests. Serum creatinine and blood urea were significantly reduced in all of the test groups as compared with EG-treated rats (p<0.001, Figures 2 and 3). RKW was comparable to normal control and significantly lower in animals treated with *B. pinnatum *than the disease control animals.( p<0.001, Figure 2)

There was significant reduction in number of crystal deposition with *B. pinnatum *(p<0.001, Figure 4). Histopathological examination of kidney showed reduced renal damage as suggested by less degeneration of epithelial lining and tubular dilatation in all the *B. pinnatum-*treated rats (Figure 5)

## Discussion

In the present study, EG-induced renal calculi model in male Wistar rats were used. Wistar rats were selected because their urinary system resembles with that of human. In addition, oxalate metabolism and excretion is almost similar to human (Khan SR and Glenton P, 1995 ▶; Vermeulen, C.W, 1962). According to previous studies, renal crystal deposition is significantly less in female than male rats (Khan SR, 1997 ▶; Otnes B, 1997 ▶; Atmani F and Khan SR, 2000 ▶). 

On administration, EG gets absorbed rapidly and metabolized into glycolic acid, glyoxylic acid, and finally converted into glycolate and oxalate by coupling of oxidation- reduction reactions (Atef M and Al-Attar, 2010 ▶). Glycolic acid oxidase and lactate dehydrogenase are enzymes involved in its metabolic pathways. The process of CaOx renal stone formation is started from calcium phosphate plaque. It is located in interstitium which is known as Randall’s plaque. Oxalate is attracted to cations to form insoluble CaOx and soluble magnesium oxalate salts. 

CaOx salt deposits on Randall’s plaque. Initially calcium oxalate monohydrate (COM) crystal attaches to renal papilla which leads to epithelial damage causing aggregation of crystals (Kuo RL et al., 2003; Kim SC et al., 2005 ▶). Subsequently, hyperoxaluria leads to nucleation, crystal growth and further aggregation, and ultimately retention in renal tubules (Khan SR et al., 1982 ▶). Administration of aqueous extract of *B. pinnatum *significantly reduced urine oxalate level. Many herbal drugs can affect metabolic pathways and activities in liver. *A. lanata* showed the reduction in oxalate production that was directly linked with reduced activities of enzymes - glycolic acid oxidase in liver and lactate dehydrogenase (Robertson WG and Peacock M, 1980 ▶). Reduction in urine oxalate by ABP may be the result of alteration in metabolism of EG that may have reduced the production of oxalate. 

Earlier report showed that CaOx crystals increased lipid peroxidation and oxidative stress to renal tissue and caused renal damage and brush border shedding by reacting with polyunsaturated fatty acids in the cell membrane (Sumathi R et al., 1993 ▶; Khan SR and Thamilselvan S. 2000 ▶). In addition, formation of calculi in EG groups leads to obstruction in outflow of urine which ultimately decreases glomerular filtration rate. As a result of cellular damage and outflow obstruction, nitrogenous waste products such as urea and creatinine accumulate in EG treated rats (Ghodkar PB, 1994 ▶). Treatment with ABP significantly decreased CaOx crystal deposition, RKW, serum creatinine, and blood urea. One *in vitro* study showed that *B. pinnatum *reduces size of calcium oxalate monohydrate crystals and promotes the formation of calcium oxalate dehydrate crystals which are less damaging to the epithelial lining of urinary tract (Fauzia Y et al., 2011 ▶). This could be the reason for significant reduction of CaOx crystal deposition.

Moreover, *B. pinnatum *contains numerous phytochemicals such as flavonoid glycosides, like quercitrin, kamferol, alkaloids, carotenoids, and saponin. Previous study showed that quercetin has nephroprotective effect on cadmium-induced nephrotoxicity (Morales AI et al., 2006 ▶). Flavonoid prevents supersaturation of calcium oxalate and decreases CaOx crystal deposition in renal tubules (Rad AK et al., 2011 ▶). 

This antioxidant property of ABP may also contribute to an improvement in serum creatinine and blood urea by preventing lipid peroxidation-induced oxidative stress. Our study has several limitations. We did not evaluate enzyme activity involved in formation of oxalate, urine substrates for composition of stone, and molecular mechanism involved in antiurolithiatic effect of extract. However, our study has given preliminary data for supporting use of *B. pinnatum* extract in urolithiasis and giving scientific evidence to folk practices. Future studies can be planned with active ingredient and improved formulations for oral use that has better bioavailability. Our study concludes that aqueous extract of *B. pinnatum *has treatment and preventive action in a preclinical model of urolithiasis. 
